# Model‐based evaluation of the interaction between ritonavir‐boosted atazanavir and rifampicin in Ugandan adults with HIV

**DOI:** 10.1002/bcp.70195

**Published:** 2025-08-12

**Authors:** Allan Kengo, Juan Eduardo Resendiz‐Galvan, Letisha Najjemba, Henry Mugerwa, Amedeo De Nicolò, Antonio D'Avolio, Shakir Atoyebi, Lubbe Wiesner, Elin M. Svensson, Catriona Waitt, Paolo Denti

**Affiliations:** ^1^ Division of Clinical Pharmacology, Department of Medicine University of Cape Town Cape Town South Africa; ^2^ Infectious Diseases Institute, College of Health Sciences Makerere University Kampala Uganda; ^3^ Joint Clinical Research Centre Research Department Kampala Uganda; ^4^ Department of Medical Sciences University of Turin Turin Italy; ^5^ Department of Women's and Children's Health University of Liverpool Liverpool United Kingdom; ^6^ Department of Pharmacy Uppsala University Uppsala Sweden; ^7^ Department of Pharmacy Radboud University Medical Center Nijmegen Netherlands

**Keywords:** antiretrovirals, drug interactions, pharmacokinetics, pharmacometrics

## Abstract

**Aim:**

Concomitant treatment of tuberculosis (TB) and human immunodeficiency virus (HIV) is complicated by drug‐drug interactions (DDI). This analysis aimed to characterize the DDI between ritonavir‐boosted atazanavir (ATV/r) and rifampicin in plasma and peripheral blood mononuclear cells (PBMC).

**Methods:**

The DERIVE study (NCT04121195) recruited Ugandan adults with HIV (not TB) on ATV/r‐based second‐line antiretroviral therapy, and collected intensive plasma and PBMC pharmacokinetic samples during four visits: (i) standard‐dose ATV/r 300/100 mg QD, (ii) same ATV/r regimen adding rifampicin 600 mg QD, (iii) doubling ATV/r to BID with rifampicin 600 mg QD and (iv) ATV/r 300/100 mg BID with rifampicin increased to 1200 mg QD. ATV/r plasma and PBMC concentrations were analysed with population pharmacokinetic modelling in NONMEM.

**Results:**

Twenty‐six participants (23 female) were enrolled, with median age and weight of 44 years and 67 kg, respectively. A two‐compartment model with an effect‐compartment effectively described atazanavir concentrations in plasma and PBMC. Rifampicin increased atazanavir clearance threefold, while decreasing its bioavailability and absorption rate. Doubling dosing frequency of ATV/r largely mitigated the interaction with rifampicin, restoring the proportion of simulated participants achieving the targeted trough atazanavir concentration of 0.014 mg/L to 99%. Rifampicin did not affect the ratio of atazanavir concentration between PBMCs and plasma.

**Conclusion:**

Metabolic induction by rifampicin accounts for the decrease in plasma exposure of ATV/r. Doubling the ATV/r dosing frequency to BID effectively mitigated this interaction. The plasma exposure of ATV/r mirrored that in PBMCs, suggesting that for these drugs, plasma concentrations provide a reliable reflection of site‐of‐action exposures.

**What is already known about this subject**

- Ritonavir‐boosted atazanavir is a WHO‐preferred protease inhibitor used as second‐line antiretroviral therapy in resource‐limited settings.
- The clinically significant drug‐drug interaction between atazanavir and rifampicin has precluded its use in patients requiring treatment for tuberculosis.

**What this study adds**

- This article uses population pharmacokinetics to describe the interaction between ritonavir‐boosted atazanavir and rifampicin in Ugandan adults with HIV. The effect of the drug‐drug interaction on atazanavir pharmacokinetic parameters is characterized in a model that is subsequently used to estimate the speed and extent of intracellular accumulation of the drug.

## 1 INTRODUCTION

Boosted protease inhibitors (bPIs), combined with an appropriate nucleoside reverse‐transcriptase inhibitor backbone, form the second‐line antiretroviral therapy (ART) regimen recommended by the World Health Organization.^1^ Ritonavir‐boosted atazanavir (ATV/r) is currently the most used bPI combination owing to its tolerability, superior potency,^2^ simplified once‐daily dosing^3^, ^4^, ^5^ and significantly reduced effects on lipid metabolism^2^, ^5^ compared with others.

Atazanavir (ATV) is rapidly absorbed and its bioavailability is enhanced by food,^3^, ^6^ but reduced by increased gastric pH.^4^ It is about 86% bound to plasma proteins (3) and a substrate of transporter proteins like p‐glycoprotein.^7^ Atazanavir is metabolized by cytochrome P450 (CYP) 3A, an enzyme it also competitively inhibits,^2^, ^3^ and is primarily eliminated by the liver through bile.^3^, ^6^ Its protein‐adjusted in vitro 90% inhibitory concentration (PA‐IC_90_) against HIV‐1 is 0.014 mg/L^3^, ^8^ and a trough plasma concentration (*C*_trough_) of 0.15 mg/L has previously been used as a target for therapeutic drug monitoring.^9^

Ritonavir was initially developed as a PI for therapeutic use but is now primarily used to enhance the pharmacokinetics of other drugs because it inhibits their metabolism.^10^, ^11^ It is an irreversible inhibitor of enzymes,^11^, ^12^, ^13^ which prevents them from metabolizing their substrates until new enzymes or cells are produced.^11^ Ritonavir is approximately 99% bound to plasma proteins and is primarily metabolized by CYP3A4^11^ and partly by CYP2D6.^12^, ^14^ Although its half‐life is only 3‐5 h,^12^ ritonavir reaches steady‐state concentrations after about 2 weeks of daily dosing, likely due to having some CYP3A4 induction properties.^13^, ^14^

Rifampicin is crucial in treating drug‐susceptible tuberculosis (TB),^15^, ^16^ a prevalent coinfection and leading cause of mortality among people living with HIV.^1^, ^16^ Currently, there is no clear guidance on its concurrent use with ATV/r due to limited clinical data on the effects of their drug‐drug interaction (DDI).^17^, ^18^ Rifampicin strongly induces CYP3A and drug transporters,^17^, ^19^ affecting ATV/r exposure.^8^ Previous investigations of DDIs between rifampicin and other bPIs had mixed results. Rifampicin caused unacceptable hepatotoxicity in patients on ritonavir‐boosted darunavir,^20^ whereas it showed mixed results in healthy volunteers and patients on ritonavir‐boosted lopinavir.^21^ Recently, higher rifampicin doses (up to 35 mg/kg) have been found to result in more favourable treatment outcomes and to be well tolerated.^22^, ^23^, ^24^

A physiologically‐based pharmacokinetic model developed by Montanha et al predicted that doubling the dosing frequency of ATV/r to 300/100 mg twice daily (BID) would effectively mitigate its interaction with rifampicin.^25^ The regimen was subsequently evaluated in the DERIVE trial, whose non‐compartmental analysis (NCA) showed that the administration of BID ATV/r with rifampicin largely restored ATV *C*_trough_ ^8^ and resulted in no cases of hepatotoxicity. In this population pharmacokinetics analysis, we aimed to further characterize the effect of the rifampicin coadministration on the pharmacokinetic parameters of ATV/r. We sought to use intracellular concentrations to characterize the distribution of ATV/r into peripheral blood mononuclear cells (PBMCs) and investigate whether rifampicin affects drug concentrations at the site of action. We also simulated the probability of achieving therapeutic target *C*_trough_ with current treatment recommendations.

## 2 METHODS

### 2.1 Study design and participants

Data were available from DERIVE (NCT04121195), an open‐label, single arm, dose‐escalation study conducted at the Joint Clinical Research Center (JCRC) in Uganda.^8^ The study enrolled adults with undetectable HIV viral load (<50 copies/mL) and on ATV/r‐based second line ART for at least 6 months. Participants were excluded if they were pregnant or breastfeeding, had coinfections like TB and hepatitis, or were taking medication known to interact with study drugs. The study was approved by the JCRC Ethics Committee (JC1819), the University of Liverpool Research Ethics Committee (Ref 5802) and the Uganda National Council for Science and Technology (HS2685). All participants provided voluntary written informed consent.

### 2.2 Sample collection and drug quantification

Pharmacokinetic sampling was conducted over four visits. During visit 1 (day 7 after recruitment), participants were still on ATV/r 300/100 mg once daily (QD). Afterwards, rifampicin 600 mg QD and dolutegravir 50 mg twice daily (BID) were added to the regimen and visit 2 sampling was carried out on day 21. The dosing frequency of ATV/r was then increased to BID, with further sampling conducted during visit 3 (day 28). The rifampicin dose was increased to 1200 mg once daily (QD) for 1 week. Sampling for visit 4 was conducted on day 35, prior to discontinuing rifampicin and resuming the standard QD ATV/r dose. Dolutegravir was maintained for an additional 2 weeks. Blood samples were collected for plasma separation at pre‐dose, and 0.5‐, 1‐, 2‐, 4‐, 6‐, 8‐, and 12‐h postdose during all visits, with an extra 24‐h sample during visit 1. Separate trough samples for intracellular PBMC concentration assays were collected at visits 1, 3 and 4 and at 12 h postdose during visit 2.

Blood samples were centrifuged and stored at −80 °C prior to shipment and assay at the University of Cape Town, where drug concentrations were measured using high‐performance liquid chromatography with tandem mass spectrometry (HPLC MS/MS). The lower limits of quantification (LLOQ) were 0.030 mg/L for atazanavir and 0.005 mg/L for ritonavir.^8^ A separate blood sample was collected in a cell preparation tube for subsequent PBMC isolation by density gradient centrifugation.^26^ Isolated PBMCs were stored at −80 °C and transferred to the University of Turin in Italy. PBMCs were counted using a previously described turbidimetric method,^27^ and intracellular atazanavir and ritonavir concentrations assayed using HPLC MS/MS^26^, ^28^ (LLOQ 0.015 mg/L).^26^, ^29^

Additional atazanavir data (without ritonavir) were available from the ACTG A5213 study, which enrolled healthy adult volunteers in the United States.^30^ Atazanavir was administered with or without rifampicin in three periods: 300 mg BID for 8 days (period 1), 300 mg BID with rifampicin 600 mg QD for 11 days (period 2) and 400 mg BID with rifampicin 600 mg QD for 8 days (period 3).^30^ Pharmacokinetic sampling was conducted at the end of each period, with blood collected 15 min predose and at 1‐, 2‐, 3‐, 4‐, 5‐, 6‐, 8‐, 10‐, 12‐, and 24‐h postdose. Plasma samples were analysed at the University of Alabama using HPLC with UV detection (LLOQ 0.025 mg/L).^30^

### 2.3 Pharmacokinetic analysis

Population pharmacokinetic analysis was done in NONMEM v7.5.1^31^ using first‐order conditional estimation with eta‐epsilon interaction. Perl‐speaks‐NONMEM^32^ and Pirana were used during the modelling process while the Xpose4 package in R via RStudio was used for model diagnostics.^33^

Atazanavir and ritonavir pharmacokinetic models were separately developed using DERIVE data. We tested one‐ and two‐compartment disposition models with linear elimination, and delayed absorption modelled by a lag time or series of transit compartments. PBMC concentrations were modelled using a hypothetical effect compartment connected to the central compartment of the plasma model. An equilibration half‐life (t_1/2_) and a pseudopartition coefficient (PPC) were used to parameterize the rate of drug entry into the PBMC compartment and its accumulation ratio between the PBMC and plasma, respectively (Figure S1).

Considering each administered dose as a separate occasion, we tested log‐normally distributed between occasion variability (BOV)^34^ on all absorption parameters. Between‐subject (BSV)^35^ and between‐visit (BVV) variability were also tested on clearance to describe its variance across individuals and study visits, respectively. Residual unexplained variability (RUV) was modelled with a combined additive and proportional error model,^35^ fixing the additive error to at least 20% of the corresponding LLOQ. Concentrations below LLOQ (BLQ) concentrations were included by imputing 50% of the LLOQ and inflating their additive component of the RUV by LLOQ/2.^36^

Total body weight or fat‐free mass (FFM)^37^ were tested to apply allometric scaling^38^ to all clearance and volume parameters. Various approaches were tested to model rifampicin and ATV/r interactions. Ritonavir and rifampicin AUCs were evaluated as continuous covariates and study visits/rifampicin dosing regimen as categorical covariates. A joint atazanavir‐ritonavir model like that by Zhang et al^18^ was also tested. A covariate was retained in the model if its addition resulted in a drop in objective function value (ΔOFV) of more than 3.84, which was considered significant at *P* < 0.05.^35^

Model performance was also assessed using goodness‐of‐fit plots, a visual predictive check (VPC) and sampling importance resampling.^39^ Monte Carlo simulations were used to estimate the probability of achieving available atazanavir treatment targets (*C*_trough_ higher than 0.014 mg/L^3^ or 0.15 mg/L^9^). Exposure was estimated for different dosing regimens using a reference cohort of 1225 in‐silico individuals based on demographic data from previous African HIV/TB studies.^40^ To evaluate the role of ritonavir in the interaction with rifampicin, we evaluated the final ATV/r model with the additional ATV‐only data from the A5213 study, allowing re‐estimation of some absorption parameters.

## 3 RESULTS

### 3.1 Study participants and data

The study enrolled 26 participants (88% female), with median age and weight of 44 years and weight 67 kg, respectively. All participants were taking lamivudine and 17 (65%), eight (31%) and one (4%) were also on tenofovir disoproxil fumarate (TDF), zidovudine and abacavir, respectively, as shown in Table 1. Concentrations in 857 plasma samples were included in the analysis, of which 28 (3%) atazanavir and 20 (2%) ritonavir samples were below the quantification limit. External atazanavir data (355 samples) were available from the ACTG A5231 study, which enrolled 13 (eight male) participants with median age and weight of 30 years and 75 kg, respectively (Table S2).

**TABLE 1:** Participant baseline characteristics.

Characteristic^a^	Value
Participants, n	26
Female, n	23 (88)
Black African, n	23 (100)
Participants living with HIV, n	26 (100)
Age, years	44 (23‐61)
Weight, kg	67 (50‐75)
Fat‐free mass, kg	41.0 (37.9‐41.9)
Body mass index, kg/m^2^	26.1 (19.9‐31.6)
Height, m	1.59 (1.48‐1.86)
ART baseline drug, n	
TDF	17 (65)
AZT	8 (31)
ABC	1 (4)

Abbreviations: ABC, abacavir; AZT, zidovudine; BMI, body mass index; TDF, tenofovir disoproxil fumarate.

^a^ The characteristics are presented as number (%) or median (range).

### 3.2 Atazanavir model

**FIGURE 1:**
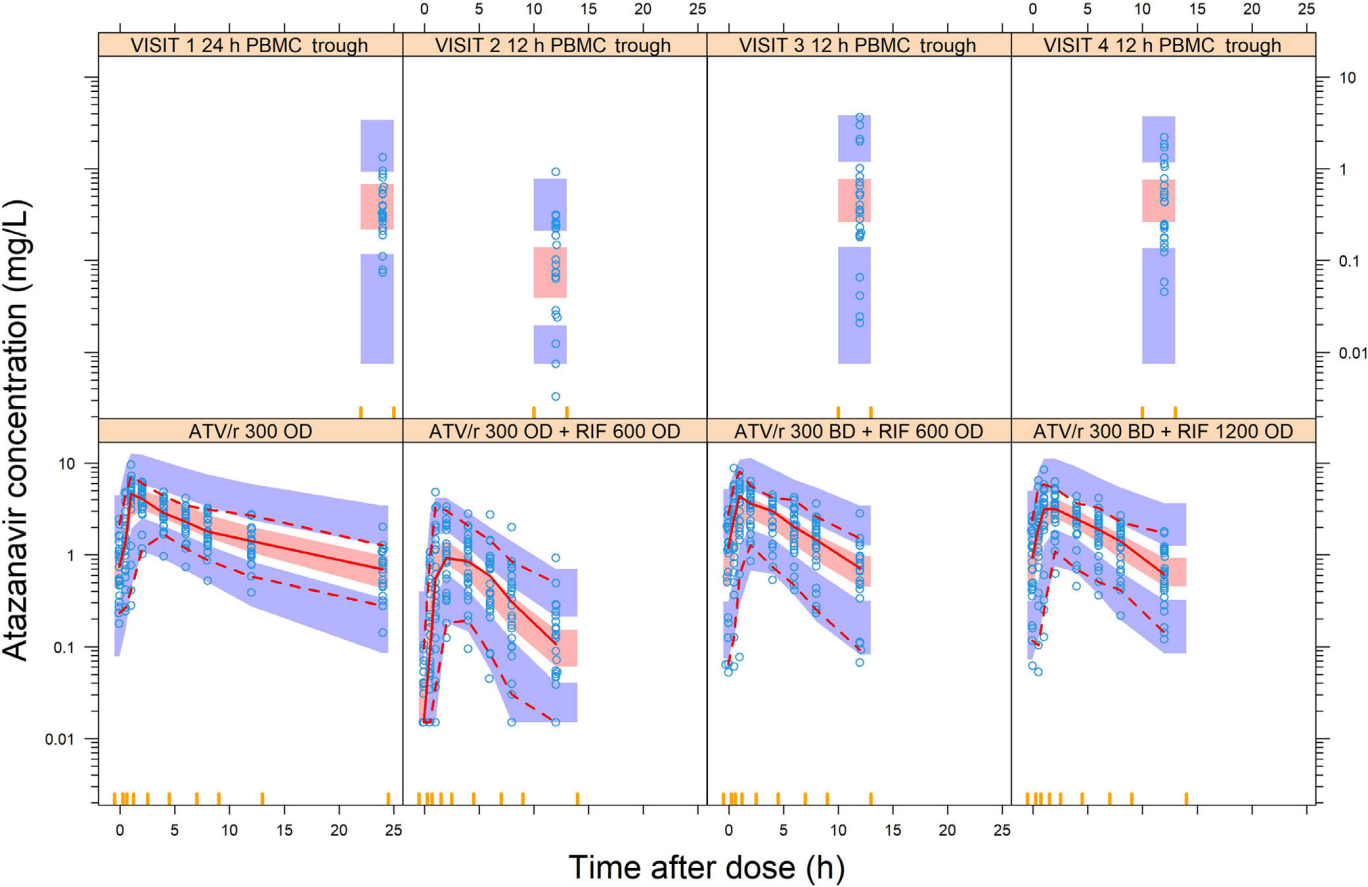
Visual predictive check of plasma (bottom) and intracellular (top) atazanavir concentrations *vs* time. The red solid and dashed lines represent the 5th, 50th and 95th percentiles of the observed data (open blue circles), while the shaded areas represent the model‐predicted 95% confidence intervals for the same percentiles. ATV/r, ritonavir‐boosted atazanavir; PBMC, peripheral blood mononuclear cells.

Atazanavir plasma data were best described by a two‐compartment model (ΔOFV = −202, *P* < 0.001, compared to one‐compartment model) with transit compartment absorption (ΔOFV = 21, *P* < 0.001, compared to lag time) and first‐order elimination. The typical (95% confidence interval) atazanavir clearance was 7.57 L/h (6.30‐9.03) when standard dose ATV/r was given OD without rifampicin. A VPC of the model fit is shown in Figure 1.

### 3.3 Ritonavir model

**FIGURE 2:**
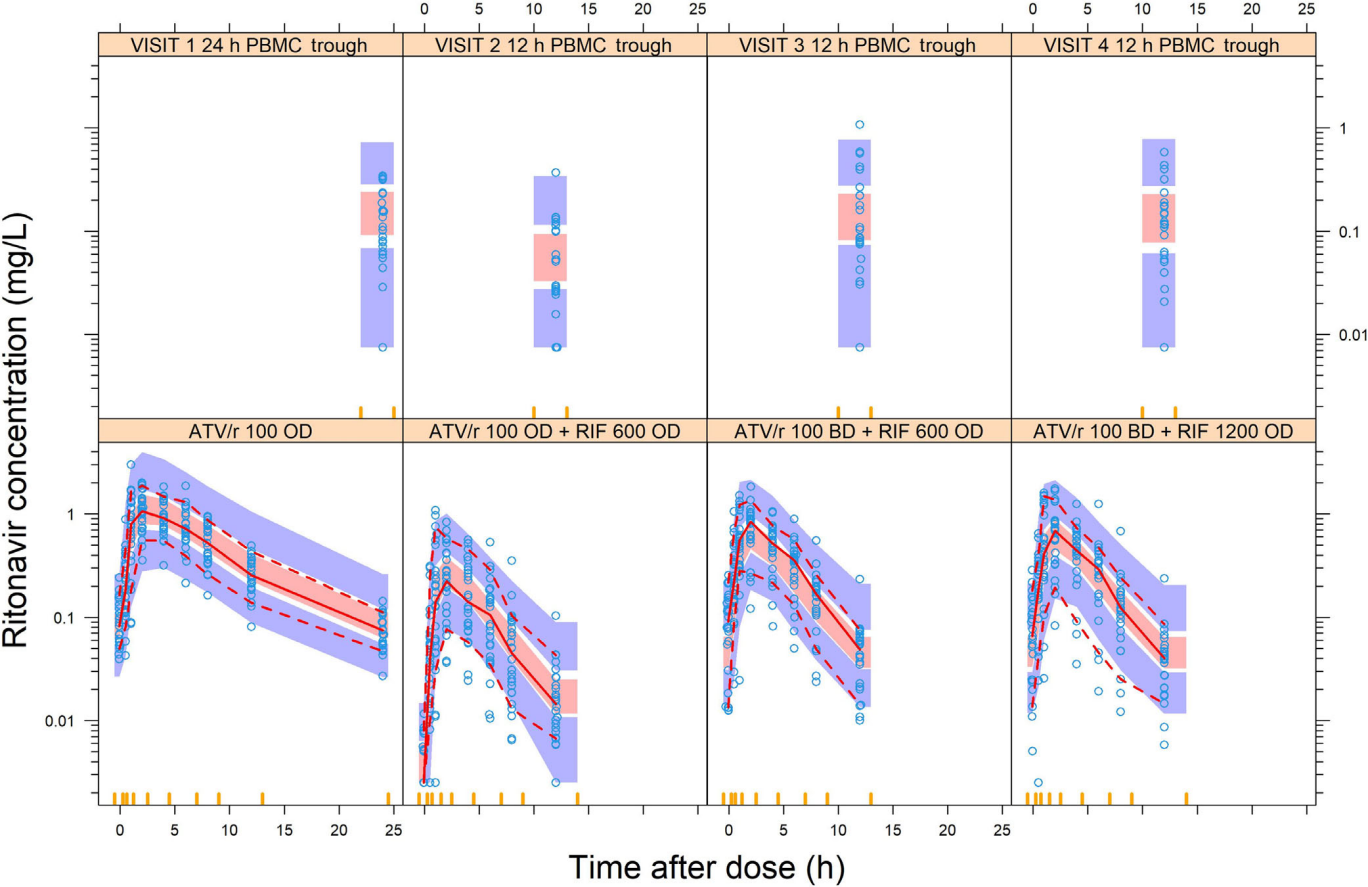
Visual predictive check of plasma (bottom) and intracellular (top) ritonavir concentrations *vs* time the red solid and dashed lines represent the 5th, 50th and 95th percentiles of the observed data (open blue circles), while the shaded areas represent the model‐predicted 95% confidence intervals for the same percentiles. ATV/r, ritonavir‐boosted atazanavir; RIF, rifampicin; PBMC, peripheral blood mononuclear cells.

Ritonavir plasma pharmacokinetics was characterized by a two‐compartment model (ΔOFV = −271, *P* < 0.001, compared to one‐compartment) and absorption through transit compartments (ΔOFV = −52, *P* < 0.001 compared to lag time) (Figure 2). The typical clearance of ritonavir was 9.67 L/h (8.51‐11.6) when administered as ATV/r without rifampicin. Clearance and volume parameters of both drugs were allometrically scaled by FFM, and other parameter estimates are presented in Table 2.

**TABLE 2:** Table of model pharmacokinetic parameter estimates.

Parameter	Typical parameter estimates (95% CI^b^)	
	Ritonavir	Atazanavir
Clearance, CL (L/h)^a^	9.67 (8.51‐11.6)	7.57 (6.42‐9.06)
Fold‐change in CL for ATV/r QD + RIF (‐fold)	2.12 (1.95‐2.31)	3.05 (2.67‐3.45)
Fold‐change in CL for ATV/r BID + RIF (‐fold)	‐	2.03 (1.82‐2.25)
Volume of distribution (central compartment) (L)^a^	55.4 (46.3‐69.1)	77.5 (69.3‐88.7)
Inter compartmental clearance (L/h)^a^	1.56 (1.17‐2.15)	3.13 (2.34‐4.31)
Volume of distribution (peripheral compartment) (L)^a^	70.1 (39.7‐125)	42.1(26.6‐79.0)
Bioavailability, F (fraction)	1 fixed	1 fixed
Change in F for ATV/r QD + RIF (%)	−68.8 (−75.2 −58.5)	−52.5 (−62.5 −41.4)
Change in F for ATV/r BID + RIF (%)	−33.3 (−46.6 −10.9)	…
Absorption rate constant, ka (/L)	1.02 (0.864‐1.27)	6 Fixed
Change in *k*_a_ due to RIF (%)	…	−67.3 (−76.2 −53.2)
Mean absorption transit time, MTT (h)	0.483 (0.428‐0.545)	0.499 (0.429‐0.574)
Transit compartments, NN (n)	12.3 (6.64‐17.7)	10 Fixed
Additive error (plasma) (mg/L)	0.001 Fixed	0.006 Fixed
Proportional error (plasma) (%)	25.6 (24.1‐27.8)	19.8 (18.2‐21.1)
Variability (% CV)^c^		
Between subject variability in clearance	16.4 (12.3‐21.8)	27.6 (19.7‐36.7)
Between visit variability in clearance	…	17.5 (14.4‐24.5)
Between occasion variability (BOV) in *k*_a_	82.3 (67.3‐99.1)	97.9 (80.8‐125)
BOV in MTT	43.6 (37.7‐51.6)	59.4 (49.3‐73.6)
BOV in F	55.5 (48.1‐63.8)	48.2 (40.7‐53.6)
Scaling factor on BOV for unobserved dose (‐fold change)^d^	…	1.63 (1.26‐2.10)
Peripheral blood mononuclear cell (PBMC)		
Equilibration half‐life, *t*_1/2_ (h)	1.40 (1.38‐1.63)	0.963 (0.546‐1.51)
Pseudo‐partition coefficient, PPC (.)	1.68 (0.643‐1.75)	0.653 (0.538‐0.797)
Proportional error PBMC (%)	51.4 (42.9‐61.1)	74.9 (62.4‐92.0)
Additive error PBMC (mg/L)	0.003 fixed	0.003 fixed

Abbreviations: QD, once daily; BID, twice daily; ATV/r, ritonavir‐boosted atazanavir; RIF, rifampicin.

^a^ All clearance and volume parameters for atazanavir and ritonavir were allometrically scaled using fat‐free mass. The values reported here refer to a typical participant with a weight of 67 kg and fat‐free mass of 42 kg.

^b^ Parameter uncertainty was determined by sampling importance resampling to obtain the 95% confidence interval (CI).

^c^ Variability in these parameters was modelled as either between subject (BSV), between‐occasion (BOV), or between‐visit (BVV) variability. It was assumed to be log‐normally distributed and is reported here as the percent coefficient of variation (%CV) calculated by $\%\mathrm{CV}=\sqrt{\omega^{2}}\times 100$.

^d^ Multiplicative factor increasing the BOV of absorption parameters (absorption rate constant, mean transit time and bioavailability) for pre‐dose concentrations following an unobserved dose.

Rifampicin data were acceptably characterized by fitting a previously published one‐compartment model with saturation of elimination via a liver compartment.^41^, ^42^ The parameters of the model were re‐estimated and are presented in Table S1 while a VPC of the data is shown in Figure S2.

### 3.4 Interactions between drugs

There was a strong correlation between ritonavir and atazanavir clearance and absorption parameters (Figures S4 and S5). Attempts to fit a ritonavir‐based inhibition model for atazanavir led to limited improvement in fit and reduced parameter precision. Similarly, including rifampicin AUC to explain inter‐visit differences in atazanavir clearance did not provide more benefit over considering each dosing regimen as its own category.

Atazanavir: Adding rifampicin to the standard ATV/r regimen increased atazanavir clearance by 3‐fold (2.7‐3.6) (ΔOFV = −122, *P* < 0.001) and reduced its bioavailability and absorption rate by 53%^40^, ^62^ (ΔOFV = 47, *P* < 0.001) and 67% (78‐56) (ΔOFV = 19, *P* < 0.001), respectively. Doubling the dosing frequency of ATV/r to BID with standard dose rifampicin restored atazanavir bioavailability and reduced the rifampicin induction of its clearance to 2‐fold (1.8‐2.3). There was no significant effect of increasing rifampicin dose on atazanavir clearance or bioavailability, and TDF affected neither atazanavir clearance (ΔOFV = 0.64, *P* = 0.424) nor bioavailability (ΔOFV = 2.66, *P* = 0.103).

Ritonavir: Rifampicin increased ritonavir clearance by 2‐fold (1.95‐2.31) (ΔOFV = −163, P < 0.001) and decreased its bioavailability to 31%^18^, ^25^, ^26^, ^27^, ^28^, ^29^, ^30^, ^31^, ^32^, ^33^, ^34^, ^35^, ^36^, ^37^, ^38^, ^39^ (ΔOFV = −63, *P* < 0.001). Doubling the dosing frequency of ATV/r partially restored ritonavir bioavailability to 66% (54‐89) (ΔOFV = −8, *P* = 0.005) and increasing the rifampicin dose had no further effect on ritonavir pharmacokinetics.

### 3.5 PBMC concentrations

The atazanavir PBMC concentrations were linked to the central plasma compartment by a distributional equilibration *t*_1/2_ of 0.963 h (0.546‐1.51) and plasma‐to‐PBMC PPC of 0.653 (0.538‐0.797), neither of which were affected by addition of rifampicin to the regimen. For ritonavir, the *t*_1/2_ and PPC were 1.40 h (1.38‐1.63) and 1.68 (0.643‐1.75), respectively. The other model parameters are presented in the Table 2.

### 3.6 Additional atazanavir data

The final atazanavir model, developed with DERIVE data, was applied to the A5231 dataset. We made the following adjustment to our model to account for the differences between the two studies. Firstly, A5231 was found to have slower absorption compared to DERIVE (MTT was 2.5‐fold [2.3‐3.2] longer, ΔOFV = −81, *P* < 0.001). Additionally, the clearance of atazanavir in all PK visits of A5231 was 2‐fold (1.9‐2.3) higher compared to when ATV/r was given without rifampicin in visit 1 of the DERIVE study (ΔOFV = 93, *P* < 0.001). Finally, a significant 55%^64–46^ reduction in atazanavir bioavailability was observed when rifampicin was coadministered with atazanavir (periods 2 and 3) (ΔOFV = −63, *P* < 0.001). A VPC and other model parameter estimates are presented in Figure S6 and Table S3.

### 3.7 Simulations of atazanavir trough plasma concentrations

**FIGURE 3:**
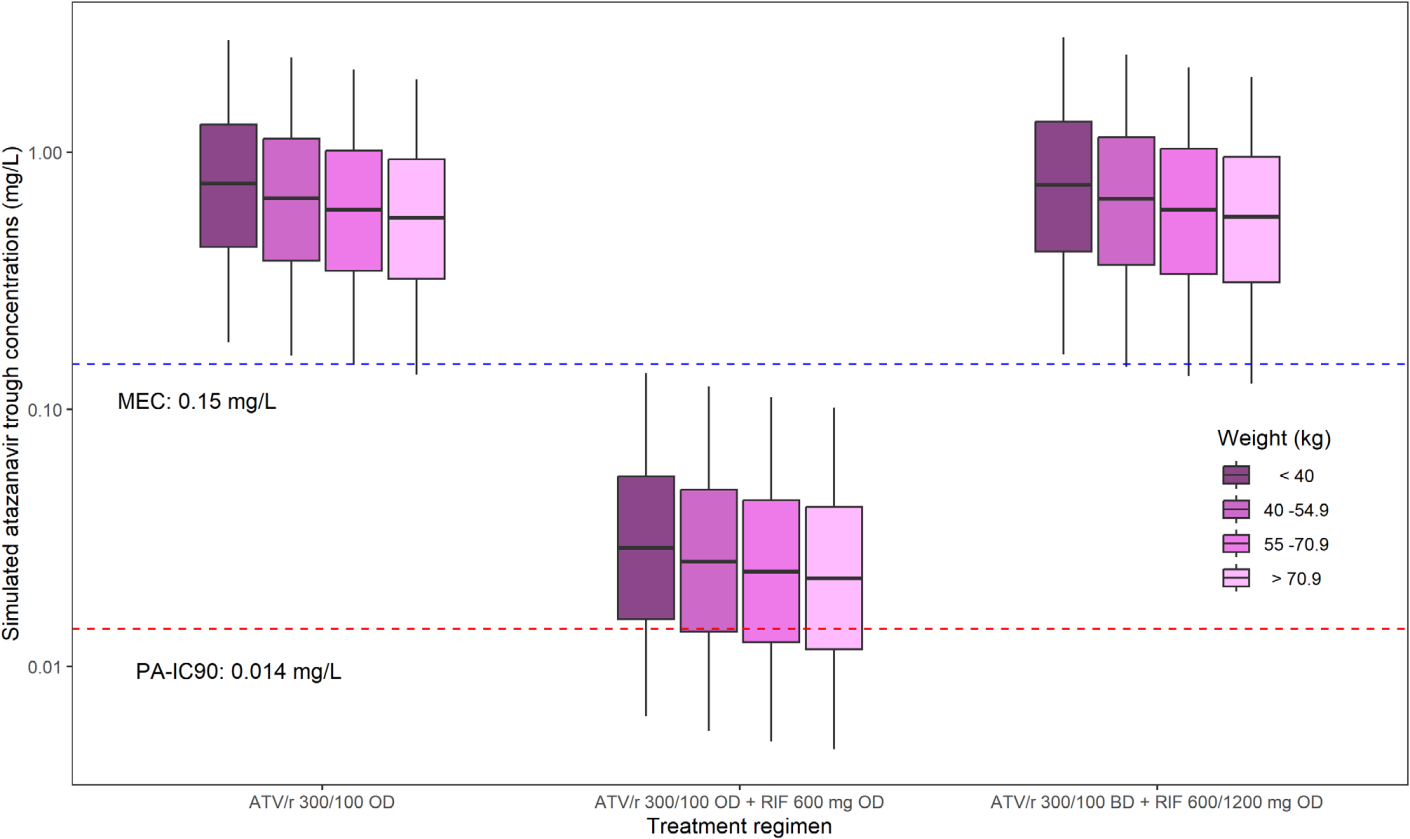
Simulated trough plasma atazanavir concentrations of participants (stratified by WHO weight bands) in different dosing scenarios. The dashed lines represent atazanavir target trough concentrations of 0.15 mg/L (blue) and 0.014 mg/L (red), respectively. The first group of boxes represents the trough concentrations achieved during ATV/r OD. The boxes in the middle are the trough concentrations reached by the simulated individuals when rifampicin 600 mg OD is added to ATV/r OD. The third dosing scenario represents trough concentrations achieved when ritonavir boosted atazanavir is given twice daily with both 600 and 1200 mg of rifampicin. ATV/r, ritonavir‐boosted atazanavir; BD, twice daily; MEC, minimum effective concentration; OD, once daily; PA_IC90, protein adjusted 90% inhibitory concentration; RIF, rifampicin;.

**FIGURE 4:**
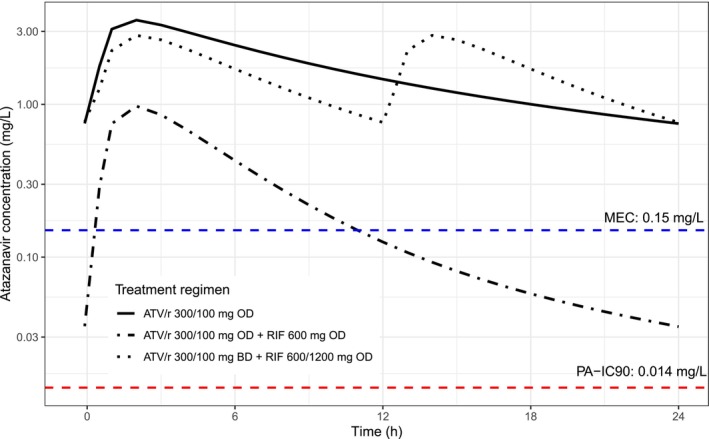
Atazanavir concentration *vs.* time profiles of the typical 61 kg individual during the three different dosing scenarios. The solid curve represents exposure when the ATV/r is given alone in the standard regimen. The dash‐dot and the dotted lines represent exposure when standard and doubled the frequency doses of ATV/r are administered with rifampicin, respectively. The lower and upper horizontal dashed lines represent the protein‐adjusted 90% inhibitory concentration (PA IC90) and commonly used target trough atazanavir concentration (MEC), respectively.

Figure 3 and Figure S3 present a summary of the predicted atazanavir *C*_trough_ and are under the curve (AUC), respectively, attained when ATV/r was dosed QD without rifampicin and then QD or BID with rifampicin. When ATV/r was administered QD alone, all (100%) simulated individuals attained a *C*_trough_ greater than PA‐IC_90_ (0.014 mg/L) and 95.4% also attained the higher 0.15 mg/L target. When given with rifampicin, the proportion of participants whose *C*_trough_ was above the PAIC_90_ and 0.15 mg/L dropped to 72.8% and 2.83%, respectively. In the third scenario, when the dosing frequency of ATV/r was doubled in the presence of standard dose rifampicin, the proportion of simulated participants who attained a *C*_trough_ greater than the PAIC_90_ and 0.15 mg/L were restored to 99% and 94%, respectively. Figure 4 shows the simulated atazanavir exposure of the typical individual in the three dosing scenarios.

## 4 DISCUSSION

In this pharmacokinetic study, we modelled the effect of rifampicin on ATV/r pharmacokinetics in Ugandan HIV patients without TB. Rifampicin reduced atazanavir and ritonavir exposures by inducing their clearance and reducing bioavailability. Our simulations showed that doubling ATV/r dosing from QD to BID restores atazanavir exposure, achieving a comparable *C*_trough_ to standard ATV/r QD without rifampicin, which was confirmed by PBMC observations. No stronger interaction was observed with the higher rifampicin dose.

These findings are consistent with previous studies showing that rifampicin reduces the exposure of PIs by induction of CYP3A enzymes and transporters,^3^, ^12^, ^18^, ^30^, ^43^ through activation of the pregnane X receptor.^17^, ^44^, ^45^, ^46^ Although more complex models like a ritonavir‐inhibition ATV model were explored, they did not provide more meaningful benefit over using visit or drug regimen as categorical covariates. This is likely because the interaction involves a shared clearance pathway for both drugs, making it difficult to distinguish causality from correlation, as illustrated in Figures S4 and S5. Moreover, both drugs also inhibit their own metabolism and were administered at a constant ratio in all participants, further limiting our ability to characterize the ATV‐ritonavir DDI within this dataset.

Our model found rifampicin to have a more pronounced effect on atazanavir clearance than ritonavir, but it caused greater reduction in ritonavir bioavailability. This is likely due to rifampicin's induction of gut CYP3A^13^, ^45^ and efflux transporters,^46^, ^47^, ^48^ which can delay drug absorption, as previously reported for atazanavir^7^ and digoxin.^47^ When ATV/r dosing frequency was increased to BID, atazanavir's bioavailability was restored. Interestingly, this was not observed with the additional A5231 ATV‐only data, suggesting that doubling the dosing frequency of ritonavir, and possibly not ATV, has a protective effect on ATV bioavailability when given with rifampicin. Moreover, we observed a twofold increase in atazanavir clearance compared to the DERIVE study, likely due to the absence of ritonavir's strong inhibitory effect.^49^ In our study, a higher rifampicin dose did not further induce atazanavir clearance, possibly due to near‐maximal enzyme induction at the standard dose.^50^, ^51^

Differences in rifampicin's effect on atazanavir and ritonavir may stem from their distinct metabolic pathways and interactions with CYP enzymes. Ritonavir's interaction with less inducible CYP2D6^45^, ^52^ may also make it less affected by rifampicin. Additionally, it binds differently to the CYP3A4 active site compared to atazanavir, leading to either metabolism or inhibition.^11^, ^13^ This could make ritonavir less available for metabolism, resulting in less induction by rifampicin.

A two‐compartment model provided a better fit for both drugs, with clearance estimates like previous studies.^53^, ^54^ As previously reported,^55^ no significant interaction between TDF and ATV/r was observed in our study, contrasting with reports in pregnant women^50^ and pre‐treated patients.^51^ Despite inhibiting p‐glycoprotein,^56^ TDF's interaction with atazanavir may be less significant in the presence of ritonavir, a stronger inhibitor.

Our model estimates of the intracellular accumulation of atazanavir and ritonavir are consistent with previous reports.^28^ The accumulation *t*_1/2_ between plasma and PBMCs was estimated to be around 1 h, differing from previously assumed instantaneous equilibration.^57^, ^58^ Despite having limited PBMC sampling points, the precision of the *t*_1/2_ estimate was confirmed through sensitivity analysis. This delay may reflect the time needed for drug molecules to cross cellular membranes. These findings suggest that plasma concentrations reliably indicate intracellular levels and drug activity at the PBMC site of action.

The study had several limitations. Participants were required to weigh between 50 and 75 kg, excluding more severely ill TB patients, and the sample was predominantly female, potentially limiting the detection of previously reported sex‐related differences.^59^, ^60^ Another study (NCT03923231) has been carried out to explore this interaction in participants with more extreme demographics. The fixed‐dose ATV/r regimen limited our ability to describe ATV‐RTV interaction with our data alone prompting the use of additional ATV‐only data. The rifampicin dose was lower than the proposed 35 mg/kg,^61^ which may affect results generalizability. Rifampicin was also administered alone, excluding other anti‐TB drugs like isoniazid that may also affect CYP enzymes.^62^ Finally, intracellular ATV/r accumulation estimates were based on limited sampling and require further validation with more intensive data collection.

In conclusion, our findings provide insights into the mechanisms underlying the DDI between rifampicin and ATV/r, complementing previous non‐compartmental analyses.^8^ We also demonstrated that rifampicin does not affect the intracellular accumulation of atazanavir, suggesting plasma levels accurately reflect drug activity at the site of action. Monte Carlo simulations support the suitability of BID dosing of ATV/r to overcome the DDI with rifampicin in HIV patients, consistent with safety data from the DERIVE trial.^8^ The concurrent use of ATV/r‐based ART and rifampicin‐containing anti‐TB regimens in individuals with HIV and TB should be further evaluated.

## AUTHOR CONTRIBUTIONS

A.K. drafted the manuscript and C.W. and P.D. designed the research. A.K., J.E.R.G., L.N., H.M., A.D.N., A.D.A., S.A., L.W., E.M.S. and P.D. performed the research and analysed the data. All authors reviewed and agreed on the final version of the manuscript.

## CONFLICT OF INTEREST STATEMENT

The authors do not have any conflict of interest to declare.

## Supporting information


**Figure S1.** Schematic representation of the final model. The mean transit time (MTT) is the time the drug takes to traverse the series of transit compartments (NN) during its absorption, *k*_a_ is the absorption rate constant, PBMC is the peripheral blood mono nuclear cell, KE0 is the drug plasma‐PBMC equilibration rate constant, which describes how soon the change in plasma is reflected in the PBMC, and PPC is the pseudo‐partition coefficient, which represents the ratio of drug in PBMC to the plasma. From the central compartment the drug equilibrates to a peripheral compartment with an intercompartmental clearance (*Q*) and peripheral volume (Vp). *K*_e_ is the elimination constant rate based on clearance (CL) and central volume (*V*).
**Table S1.** Table of rifampicin model pharmacokinetic parameter estimates.
**Figure S2.** Visual predictive check of plasma rifampicin concentrations *vs* time. The red solid and dashed lines represent the 10th, 50th and 90th percentiles of the observed data (open blue circles), while the shaded areas represent the model‐predicted 95% confidence intervals for the same percentiles. ATV/r, ritonavir boosted atazanavir; RIF, rifampicin; OD, once daily; BD, twice daily.
**Figure S3.** Simulated atazanavir plasma area under the curve (AUC) of participants (stratified in weight bands) in different atazanavir dosing scenarios. The dots represent the ATV AUC observed in the study. ATV/r, ritonavir boosted atazanavir; RIF, rifampicin; OD, once daily; BD, twice daily.
**Figure S4.** Correlation matrix of unexplained variabilities in (A) clearance, (B) bioavailability, (C) absorption and (D) area under the curve from time 0 to infinity of atazanavir, ritonavir and rifampicin. Only variability from the dosing occasion associated with the observed dose on the study visit was included.
**Figure S5.** Correlation matrix of clearance and bioavailability of atazanavir, ritonavir and rifampicin. Only parameters from the dosing occasion associated with the observed dose on the study visit were included.
**Table S2.** Participant characteristics of the A5231 study.
**TABLE S3.** Table of atazanavir model pharmacokinetic parameter estimates from the ACTG A5231 data.
**Figure S6.** Visual predictive check of plasma atazanavir concentrations *vs* time. (A) DERIVE model and (B) after making changes in bioavailability (due to rifampicin) and clearance (due to absence of ritonavir inhibition). The atazanavir model developed with DERIVE study data was used to describe the exposures observed in the ACTG study (A5231) with factors describing the ‐fold change on absorption, bioavailability and clearance. The solid lines represent the 50th percentile of the observed data (open circles), while the shaded areas represent the model‐predicted 95% confidence intervals for the percentiles. ATV, atazanavir; RIF, rifampicin; BID, twice daily; QD, once daily.

## Data Availability

The datasets generated and analysed in this study may be made available by the corresponding author upon request, subject to suitable access agreements.
