# Supplementary material for: Model‐based evaluation of the interaction between ritonavir‐boosted atazanavir and rifampicin in Ugandan adults with HIV
Source: Br J Clin Pharmacol. 2025 Aug 12;91(12):3471–81. doi: 10.1002/bcp.70195 (PMC12648368; doi:10.1002/bcp.70195)
Supplement: Supplementary file 1 — Figure S1. Schematic representation of the final model. The mean transit time (MTT) is the time the drug takes to traverse the series of transit compartments (NN) during its absorption, k a is the absorption rate constant, PBMC is the peripheral blood mono nuclear cell, KE0 is the drug plasma‐PBMC equilibration rate constant, which describes how soon the change in plasma is reflected in the PBMC, and PPC is the pseudo‐partition coefficient, which represents the ratio of drug in PBMC to the plasma. From the central compartment the drug equilibrates to a peripheral compartment with an intercompartmental clearance (Q) and peripheral volume (Vp). K e is the elimination constant rate based on clearance (CL) and central volume (V). Table S1. Table of rifampicin model pharmacokinetic parameter estimates. Figure S2. Visual predictive check of plasma rifampicin concentrations vs time. The red solid and dashed lines represent the 10th, 50th and 90th percentiles of the observed data (open blue circles), while the shaded areas represent the model‐predicted 95% confidence intervals for the same percentiles. ATV/r, ritonavir boosted atazanavir; RIF, rifampicin; OD, once daily; BD, twice daily. Figure S3. Simulated atazanavir plasma area under the curve (AUC) of participants (stratified in weight bands) in different atazanavir dosing scenarios. The dots represent the ATV AUC observed in the study. ATV/r, ritonavir boosted atazanavir; RIF, rifampicin; OD, once daily; BD, twice daily. Figure S4. Correlation matrix of unexplained variabilities in (A) clearance, (B) bioavailability, (C) absorption and (D) area under the curve from time 0 to infinity of atazanavir, ritonavir and rifampicin. Only variability from the dosing occasion associated with the observed dose on the study visit was included. Figure S5. Correlation matrix of clearance and bioavailability of atazanavir, ritonavir and rifampicin. Only parameters from the dosing occasion associated with the observed dose on the study [file BCP-91-3471-s001.docx]

**Model-based evaluation of the interaction between ritonavir-boosted atazanavir and rifampicin in Ugandan adults with HIV**

**Supplementary File**

## **Methods**

High-performance liquid chromatography with tandem mass spectrometry detection methods were developed and validated to assay rifampicin at the Division of Clinical Pharmacology, University of Cape Town. The calibration ranges were: 0.030 (lower limit of quantification, LLOQ) – 10.0 mg/L for atazanavir, 0.005 (LLOQ) – 2.50 mg/L for ritonavir (1). The calibration range for the rifampicin assay was 0.117 – 30.0 mg/L, with an inter-day accuracy of 101% – 107%. The precision, measured as coefficient of variation (%CV), ranged from 2.70% – 13.7%. A more detailed description of the method has been previously described (1,2). PBMC concentrations were assayed with a previously described validated HPLC MS/MS method (3,4). The LLOQ for both drugs were 0.015 mg/L, and the accuracy ranged from 92.7 to 103.7% (3,5).

## **Results**

**Figure S1:** Schematic representation of the final model. The mean transit time (MTT) is the time the drug takes to traverse the series of transit compartments (NN) during its absorption; k_a_ is the absorption rate constant; PBMC, peripheral blood mono nuclear cell; KE0, drug plasma-PBMC equilibration rate constant which describes how soon the change in plasma is reflected in the PBMC; 𝑃𝑃𝐶, the pseudo-partition coefficient which represents the ratio of drug in PBMC to the plasma. From the central compartment the drug equilibrates to a peripheral compartment with an intercompartmental clearance (Q) and peripheral volume (Vp). K_e_ is the elimination constant rate based on clearance (CL) and central volume (V).

**Peripheral compartment**

**k_e_ = CL/V**

**Absorption compartment**

**Transit compartments MTT, NN**

**Dose**

**k_a_**

**Central compartment**

$\text{Q}$ **/** $\text{V}$

$\text{Q}$ **/** $\text{V}\text{p}$

**PBMC**

**KE0, PPC**

**KE0**

**Table S1:** Table of rifampicin model pharmacokinetic parameter estimates

| Parameter | Typical parameter estimates (95% CI ^b^) |
| --- | --- |
| Clearance, CL (L/h) ^a, e^ | 33.0 (27.2 – 40.8) |
| Michaelis‑Menten constant, Km (L/h) | 4.90 (3.82 – 6.23) |
| Volume of distribution (central compartment) (L) | 44.7 (42.2 – 47.8) |
| Bioavailability, F (fraction) | 1 (fixed) |
| Absorption rate constant, ka (/L) | 2.40 (1.74 – 3.30) |
| Mean absorption transit time, MTT (h) | 0.502 (0.447 – 0.558) |
| Transit compartments, NN (n) | 20.3 (15.8 – 25.9) |
| Additive error (mg/L) | 0.023 (fixed) |
| Proportional error (%) | 18.6 (17.1 – 20.3) |
| Hepatic volume, V_H_ (L) | 1 (fixed) |
| Hepatic blood flow rate, Q_H_ (L/h) ^a^ | 90 (fixed) |
| Unbound fraction of rifampicin, f_u_ (fraction) | 0.2 (fixed) |
| Variability (% CV) ^c^ |  |
| Between subject variability in clearance | 27.7 (22.1 – 35.2) |
| Between occasion variability (BOV) in F | 22.0 (17.9 – 26.3) |
| BOV in ka | 104 (85.3 – 136) |
| BOV in MTT | 46.8 (40.0 – 56.8) |
| Scaling factor on BOV for unobserved dose (-fold change) ^d^ | 2.54 (1.90 – 3.32) |

Parameter estimates were obtained by fitting a previously published 1-compartment model to the DERIVE study rifampicin data.

^a^ All clearance and volume parameters for atazanavir and ritonavir were allometrically scaled using fat-free mass. The values reported here refer to a typical participant with a fat-free mass of 41 kg and total body weight of 67 kg.

^b^ Values in parentheses are empirical 95% conﬁdence intervals obtained by sampling importance resampling procedure.

^c^ Variability in these parameters was modeled as either between‑subject (BSV), between-occasion (BOV), or between‑visit (BVV) variability. It was assumed to be log-normally distributed and is reported here as the percent coefficient of variation (%CV) calculated by $\boldsymbol{\%CV=}\sqrt{\boldsymbol{\omega}^{\boldsymbol{2}}}\boldsymbol{\times100}$.

^d^ Multiplicative factor increasing the BOV of absorption parameters (ka, MTT, and BIO) for pre-dose concentrations following an unobserved dose.

^e^ Clearance calculated from maximum intrinsic clearance using the formular: $\boldsymbol{CL=}\boldsymbol{CL}_{\boldsymbol{int.max}}\boldsymbol{\times}\boldsymbol{f}_{\boldsymbol{u}}$**.** where *CL_int,max_* , maximum intrinsic clearance; *f_u_*, unbound fraction of rifampicin in plasma.

**
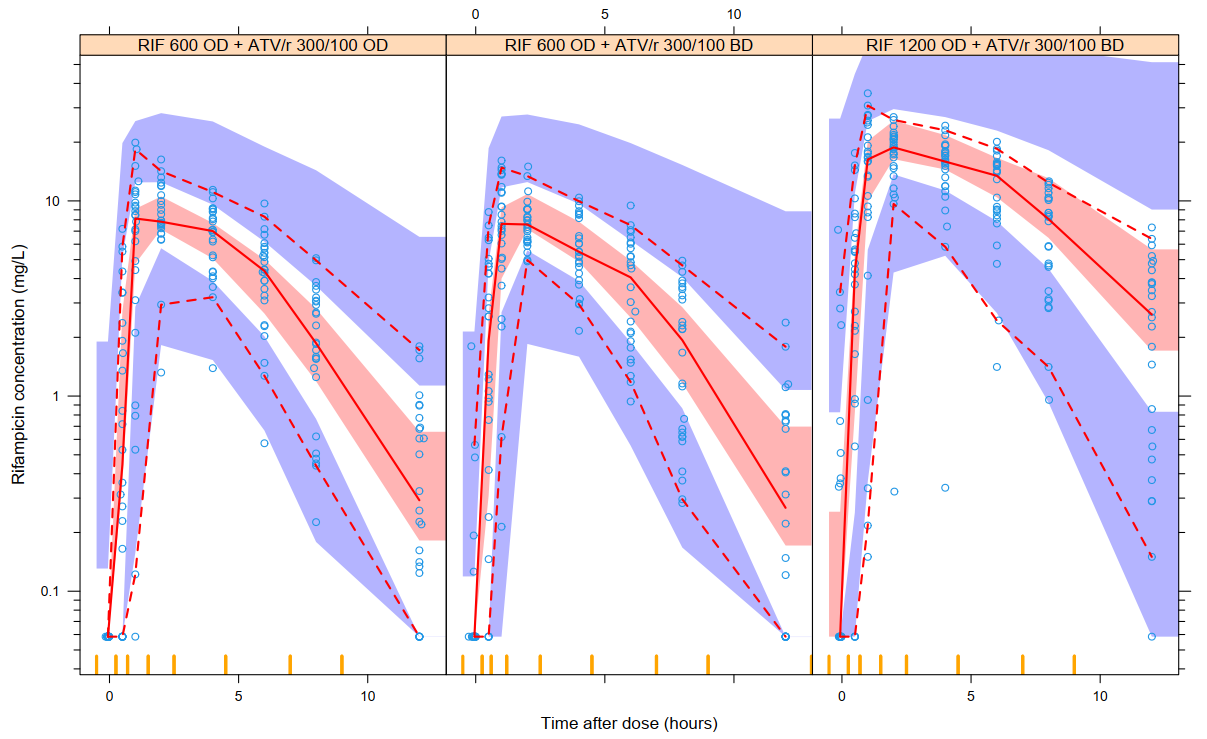
**

**Figure S2.** Visual predictive check of plasma rifampicin concentrations versus time. The red solid and dashed lines represent the 10th, 50th, and 90th percentiles of the observed data (open blue circles), while the shaded areas represent the model-predicted 95% confidence intervals for the same percentiles. ATV/r- ritonavir boosted atazanavir; RIF, rifampicin; OD, once daily; BD, twice daily.


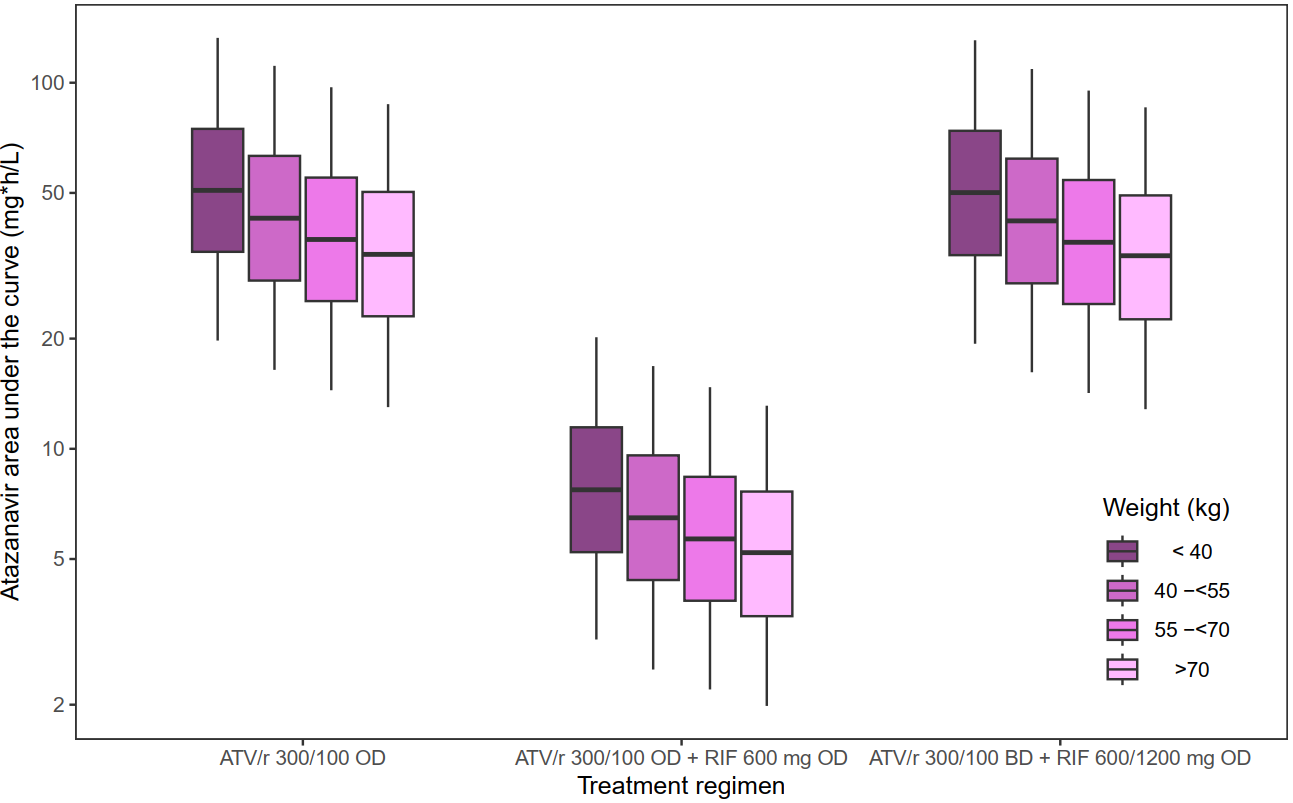


**Figure S3.** Simulated atazanavir plasma area under the curve (AUC) of participants (stratified in weight bands) in different atazanavir dosing scenarios. The dots represent the ATV AUC observed in the study. ATV/r- ritonavir boosted atazanavir; RIF, rifampicin; OD, once daily; BD, twice daily.


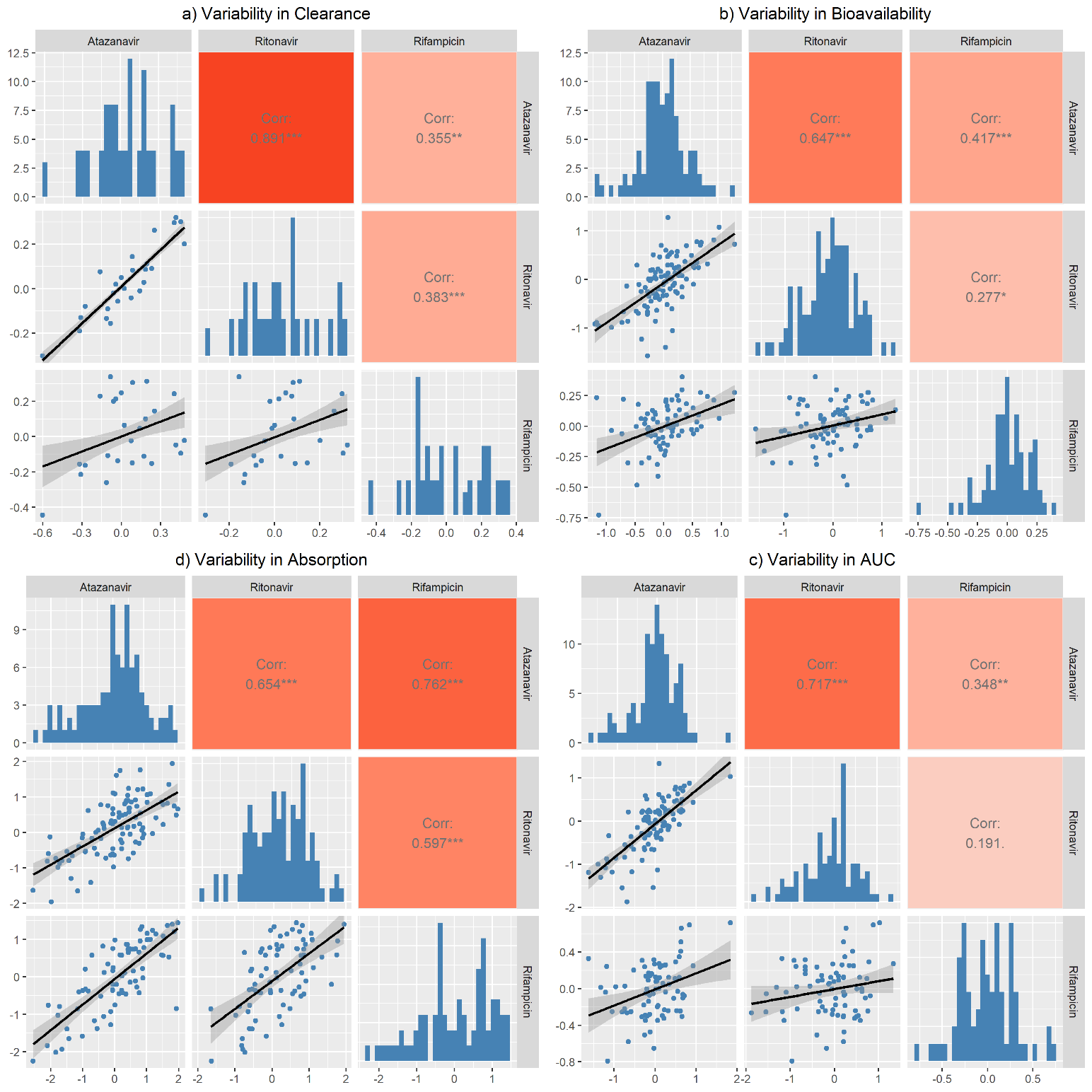


**Figure S4.** Correlation matrix of unexplained variabilities in; a, clearance; b, bioavailability; c, absorption; and d, area under the curve from time 0 to infinity) of atazanavir, ritonavir and rifampicin. Only variability from the dosing occasion associated with the observed dose on the study visit was included.


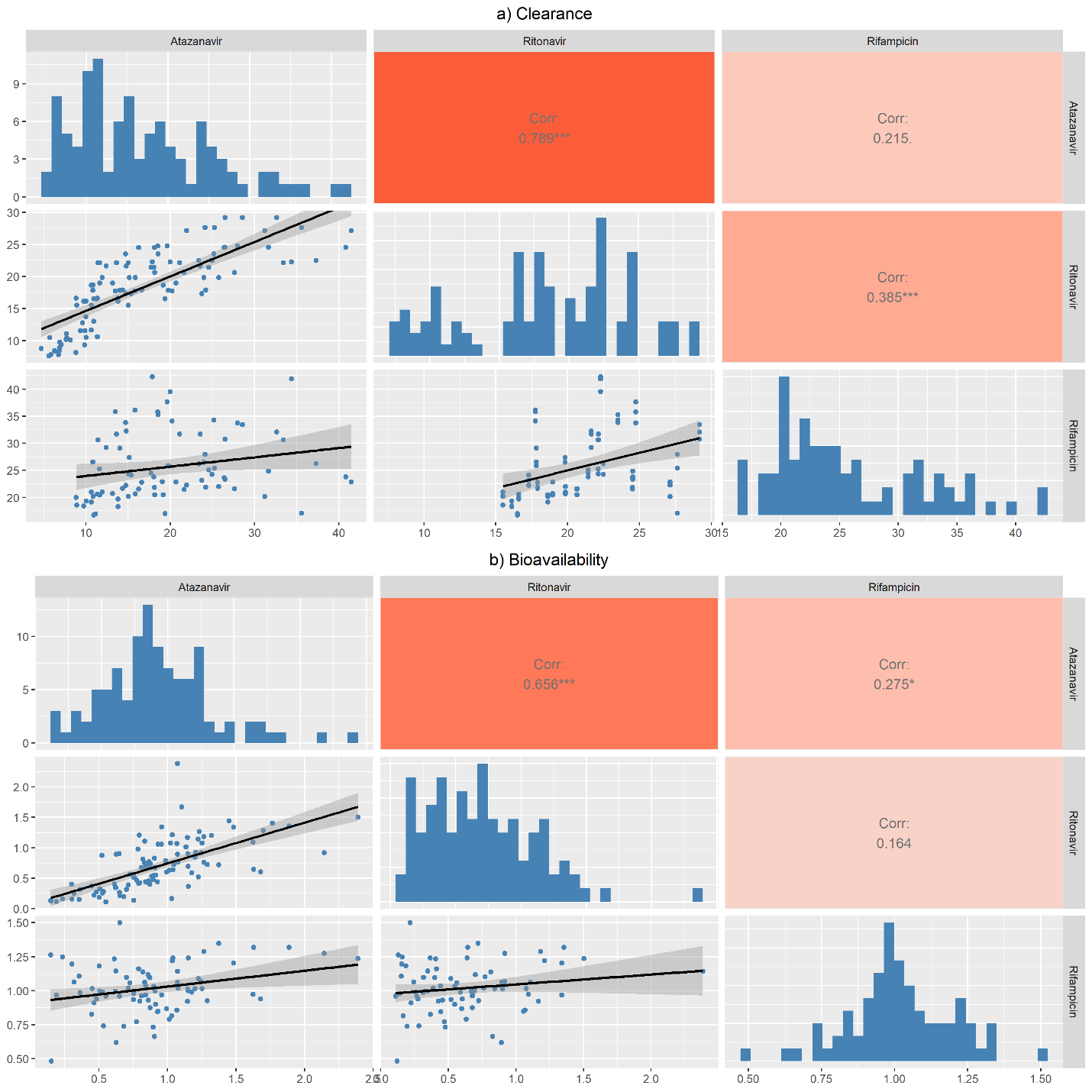


**Figure S5.** Correlation matrix of clearance and bioavailability of atazanavir, ritonavir and rifampicin. Only parameters from the dosing occasion associated with the observed dose on the study visit were included.

**Table S2:** Participant characteristics of the A5231 study

| Characteristic ^a^ | A5231 Study |
| --- | --- |
| Participants, n | 13 |
| Female, n | 5 (38) |
| Black race, n | 1 (8) |
| Participants living with HIV, n | None |
| Age, years | 30 (23 – 48) |
| Weight, kg | 75 (55 – 110) |

^a^ The characteristics are presented as number (%) or median (range).

**Table S3:** Table of atazanavir model pharmacokinetic parameter estimates from the ACTG A5231 data.

| Parameter | Typical parameter estimates (95% CI ^b^) |
| --- | --- |
| Clearance, CL (L/h) ^a^ | 7.55 (fixed) |
| Fold-change in CL due to absence of ritonavir (-fold) | **2.13 (1.88 – 2.34)** |
| Volume of distribution (central compartment) (L) | 77.3 (fixed) |
| Inter compartmental clearance (L/h) | 3.51 (fixed) |
| Volume of distribution (peripheral compartment) (L) | 48.9 (fixed) |
| Bioavailability, F (fraction) | 1 (fixed) |
| Change in F with rifampicin (%) | **-55.3 (****-64.0 – -46.2)** |
| Absorption rate constant, ka (/L) | 6 (fixed) |
| Change in ka with rifampicin (%) | -70.8 (fixed) |
| Mean absorption transit time, MTT (h) | **1.38 (1.15 – 1.61)** |
| Transit compartments, NN (n) | 10 (fixed) |
| Additive error (mg/L) | 0.005 (fixed) |
| Proportional error (%) | 18.8 (fixed) |
| Variability (% CV) ^c^ |  |
| Between subject variability in clearance | 18.8 (13.7 – 24.4) |
| Between occasion variability (BOV) in ka | 101 (fixed) |
| BOV in MTT | 48.1 (36.9 – 63.4) |
| BOV in F | 58.3 (46.4 – 73.5) |
| Scaling factor on BOV for unobserved dose (-fold change) ^d^ | 1.7 (1.10 – 2.81) |

All fixed parameters are estimates from DERIVE study population.

^a^ All clearance and volume parameters for atazanavir and ritonavir were allometrically scaled using fat-free mass. The values reported here refer to a typical participant with a fat-free mass of 41 kg and total body weight of 67 kg.

^b^ Values in parentheses are empirical 95% conﬁdence intervals obtained by sampling importance resampling procedure.

^c^ Variability in these parameters was modelled as either between‑subject (BSV), between-occasion (BOV), or between‑visit (BVV) variability. It was assumed to be log-normally distributed and is reported here as the percent coefficient of variation (%CV) calculated by $\boldsymbol{\%}CV= \sqrt{\omega^{2}} \times100$.

^d^ Multiplicative factor increasing the BOV of absorption parameters (ka, MTT, and BIO) for pre-dose concentrations following an unobserved dose.


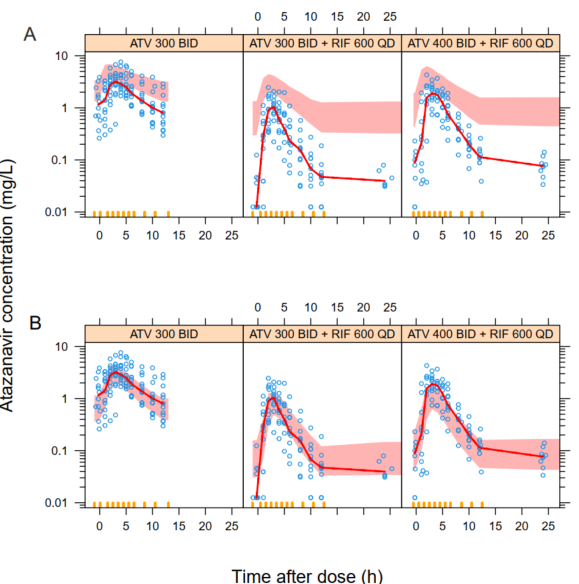


**Figure S6**. Visual predictive check of plasma atazanavir concentrations versus time: A; DERIVE model and B; after making changes in bioavailability (due to rifampicin) and clearance (due to absence of ritonavir inhibition). The atazanavir model developed with DERIVE study data was used to describe the exposures observed in the ACTG study (A5231) with factors describing the -fold change on absorption, bioavailability, and clearance. The solid lines represent the 50th percentile of the observed data (open circles), while the shaded areas represent the model-predicted 95% confidence intervals for the percentiles. ATV, atazanavir; RIF, rifampicin; BID, twice daily; QD, once daily.

ATV NONMEM control stream

**$SIZES PD=-1000 LVR=-150 LTH=-200 MAXFCN=10000000 LNP4=-150000**

**$PROBLEM VIRTUAL_ATV**

**$ABBREVIATED DERIV2=NO COMRES=2**

**$INPUT ID VISIT OCC ….**

**$DATA VIRTUAL_data_.... IGNORE=#**

**$SUBROUTINE ADVAN13 TRANS1 TOL=9 ATOL=9**

**$MODEL NCOMPARTMENTS=4 COMP=(ABS DEFDOSE)**

**COMP=(CENTRAL DEFOBSERVATION) ; COMP=(CENTRAL DEFOBSERVATION)**

**COMP=(PERIPH) COMP=(PBMC)**

**$PK**

**SMALL = 1E-6**

**; ------- BSV**

**BSVCL = ETA(1)**

**BSVV = ETA(2)**

**BSVKA = ETA(3)**

**BSVBIO = ETA(4)**

**BSVV3 = ETA(5)**

**BSVQ = ETA(6)**

**BSVV4 = ETA(7)**

**BSVQ2 = ETA(8)**

**BSVMTT = ETA(9)**

**;------for effect cmpt**

**BSVKE0 = ETA(46)**

**BSVPPC = ETA(47)**

**; ---------- BOV**

**BOVCL = 0**

**IF (OCC==1)BOVCL = ETA(10)**

**IF (OCC==2)BOVCL = ETA(11)**

**IF (OCC==3)BOVCL = ETA(12)**

**IF (OCC==4)BOVCL = ETA(13)**

**IF (OCC==5)BOVCL = ETA(14)**

**IF (OCC==6)BOVCL = ETA(15)**

**IF (OCC==7)BOVCL = ETA(16)**

**IF (OCC==8)BOVCL = ETA(17)**

**BOVBIO = 0**

**IF (OCC==1)BOVBIO = ETA(18)**

**IF (OCC==2)BOVBIO = ETA(19)**

**IF (OCC==3)BOVBIO = ETA(20)**

**IF (OCC==4)BOVBIO = ETA(21)**

**IF (OCC==5)BOVBIO = ETA(22)**

**IF (OCC==6)BOVBIO = ETA(23)**

**IF (OCC==7)BOVBIO = ETA(24)**

**IF (OCC==8)BOVBIO = ETA(25)**

**BOVKA = 0**

**IF (OCC==1)BOVKA = ETA(26)**

**IF (OCC==2)BOVKA = ETA(27)**

**IF (OCC==3)BOVKA = ETA(28)**

**IF (OCC==4)BOVKA = ETA(29)**

**IF (OCC==5)BOVKA = ETA(30)**

**IF (OCC==6)BOVKA = ETA(31)**

**IF (OCC==7)BOVKA = ETA(32)**

**IF (OCC==8)BOVKA = ETA(33)**

**BOVMTT = 0**

**IF (OCC==1)BOVMTT = ETA(34)**

**IF (OCC==2)BOVMTT = ETA(35)**

**IF (OCC==3)BOVMTT = ETA(36)**

**IF (OCC==4)BOVMTT = ETA(37)**

**IF (OCC==5)BOVMTT = ETA(38)**

**IF (OCC==6)BOVMTT = ETA(39)**

**IF (OCC==7)BOVMTT = ETA(40)**

**IF (OCC==8)BOVMTT = ETA(41)**

**;----------between visit variability**

**BVVCL = 0**

**IF (VISIT == 1) BVVCL = ETA(42)**

**IF (VISIT == 2) BVVCL = ETA(43)**

**IF (VISIT == 3) BVVCL = ETA(44)**

**IF (VISIT == 4) BVVCL = ETA(45)**

**;----------------**

**SCALE_BOV = THETA(10) ;extra bioavailability on all absorption parameters**

**IF (OBS.EQ.0) THEN**

**BOVKA=SCALE_BOV*BOVKA**

**BOVMTT=SCALE_BOV*BOVMTT**

**BOVBIO=SCALE_BOV*BOVBIO**

**ENDIF**

**; -------------- Calculation of Fat-free Mass**

**HTM = HEIGHT/100**

**IF (SEX.EQ.0) THEN ; female**

**WHSMAX=37.99**

**WHS50=35.98**

**ELSE ;males**

**WHSMAX=42.92**

**WHS50=30.93**

**ENDIF**

**HTM2 = HTM**2**

**FFM = (WHSMAX*HTM2*WT)/(WHS50*HTM2+WT)**

**FAT = WT-FFM**

**IF (FAT.LT.0) FAT = 0**

**; ------- Typical values of covariates use MEDIAN WEIGHT OF MY PPN**

**TVWT = 67**

**TVFAT = 25**

**TVFFM = 42**

**;--------- Allometric scaling and covariates**

**ALLMCL_WT = (WT/TVWT)**0.75**

**ALLMV_WT = (WT/TVWT)**

**ALLMCL_FAT = (FAT/TVFAT)**0.75**

**ALLMV_FAT = (FAT/TVFAT)**

**ALLMCL_FFM = (FFM/TVFFM)**0.75**

**ALLMV_FFM = (FFM/TVFFM)**

**;----------Allometry for liver**

**ALLMCL_WT_HEP = (WT/70)**0.75**

**ALLMV_WT_HEP = (WT/70)**

**ALLMCL_FFM_HEP = (FFM/56.1)**0.75**

**ALLMV_FFM_HEP = (FFM/56.1)**

**;-----------------------covariates**

**;;Covariate test: RTV_CL**

**MEDIAN_AUC24_RTV = 7.0923**

**RTVAUC_CL = 1 + THETA(11)*(AUC_RTV2 - MEDIAN_AUC24_RTV) ;Inter**

**VISIT_DVID = VISIT + 10*DVID**

**;;Different CL for every visit:**

**CL_VISIT = 1;no RTV**

**IF(VISIT.EQ.2) CL_VISIT = 1 + THETA(12 )**

**IF(VISIT.GE.3) CL_VISIT = 1 + THETA(13)**

**BIO_VISIT = THETA (4);**

**IF(VISIT.EQ.2) BIO_VISIT = THETA(14);v2**

**;;Different KA for visits with RIF**

**RIF_KA = 1;no RTV**

**IF(RIF.EQ.1) RIF_KA = 1 + THETA(16);Inter**

**;---------Typical values**

**TVCL = THETA(1) * ALLMCL_FFM * CL_VISIT * RTVAUC_CL;*NAT2_CL*CFZ_CL ;**

**TVV = THETA(2) * ALLMV_FFM**

**TVKA = THETA(3) * RIF_KA**

**TVBIO = BIO_VISIT**

**TVMTT = THETA(7) ;* MTT_VISIT**

**TVNN = THETA(15)**

**TVV3 = THETA(8)*ALLMV_FFM**

**TVQ = THETA(9)*ALLMCL_FFM**

**;------for effect cmpt**

**TVKE0 = THETA(17)**

**TVPPC = THETA(18)**

**;-----------Define parameters**

**CL = TVCL*EXP(BSVCL+BOVCL+BVVCL) ; CLEARANCE**

**V = TVV*EXP(BSVV) ; CENTRAL VOL.**

**KA = TVKA*EXP(BSVKA+BOVKA) ; ABS. RATE CONSTANT**

**BIO = TVBIO*EXP(BSVBIO+BOVBIO) ; BIOAVAILABILITY**

**MTT = TVMTT*EXP(BSVMTT+BOVMTT) ; MTT TIME**

**NN = TVNN ; Number of transit compartments**

**V3 = TVV3*EXP(BSVV3) ; PERIPH VOL**

**Q = TVQ*EXP(BSVQ) ; INTER COMPT CL**

**;-------for effect compartment**

**KE0 = TVKE0 * EXP(BSVKE0)**

**PPC = TVPPC * EXP(BSVPPC)**

**; re-parameterization**

**K = CL/V ;(rate constant of elimination)**

**K23 = Q/V ; (rate constant from central to peripheral 1)**

**K32 = Q/V3 ;(rate constant from peripheral 1 to central)**

**; Transit compartment absorption**

**F1=0 ;**

**KTR = (NN+1)/MTT**

**IF (NEWIND/=2.OR.EVID>=3) THEN**

**TNXD=TIME ; Time of the dose**

**PNXD=AMT ; Amount. If it's zero, the DE is deactivated.**

**ENDIF**

**TDOS=TNXD ;**

**PD=PNXD ;**

**IF(AMT>0) THEN ;**

**TNXD=TIME**

**PNXD=AMT**

**ENDIF**

**PIZZA = LOG(BIO*PD*KTR + 1E-12) - GAMLN(NN+1) ; without +0.00001, it won't work with ETAs in bioavailability**

**A_0(1) = SMALL**

**A_0(2) = SMALL**

**A_0(3) = SMALL**

**A_0(4) = SMALL ; PBMC**

**;;;--------------------------------------------------------------**

**$DES**

**C2 = A(2)/V**

**TEMPO = T-TDOS**

**KTT = 0**

**TRANSIT = 0**

**IF(PD.GT.0.AND.TEMPO.GT.0) THEN ;**

**KTT = KTR*(TEMPO)**

**TRANSIT = EXP(PIZZA+NN*LOG(KTT)-KTT)**

**ENDIF**

**DADT(1) = TRANSIT - KA*A(1)**

**DADT(2) = KA*A(1) - K*A(2) + K32*A(3) - K23*A(2)**

**DADT(3) = K23*A(2) - K32*A(3)**

**DADT(4) = KE0*(PPC*C2 - A(4)) ;A(4)**

**$ERROR**

**IPRED_P=A(2)/V**

**; DEFINE LLOQ VALUE**

**LLOQ_P = 0.03 ; DEFINE YOUR OWN LLOQ HERE**

**LLOQ_C = 0.015 ; DEFINE YOUR OWN LLOQ HERE**

**CENS_THR_P = LLOQ_P**

**PROP_P = IPRED_P*THETA(5)**

**ADD_P = THETA(6)+(CENS_THR_P*0.2)**

**IF (ICALL/=4.AND.CENS==1) THEN**

**ADD_P = ADD_P +(CENS_THR_P*0.5)**

**ENDIF**

**NO_FIT = 0**

**IF (ICALL/=4.AND.CENS==2) THEN**

**PROP_P = 0**

**ADD_P = 10000000000**

**NO_FIT = 1**

**ENDIF**

**W_P = SQRT(ADD_P**2+PROP_P**2)**

**;------PBMC**

**CC = A(4)**

**CENS_THR_C = LLOQ_C**

**IPRED_C = CC**

**PROP_C = IPRED_C * THETA(19)**

**ADD_C = THETA(20) + (0.2*CENS_THR_C)**

**IF(ICALL/=4.AND.CENS==1.AND.DVID==4) THEN**

**ADD_C = ADD_C + (LLOQ_C*0.5)**

**ENDIF**

**IF (ICALL/=4.AND.CENS==2.AND.DVID==4) THEN**

**PROP_C = 0**

**ADD_C = 10000000000**

**NO_FIT = 1**

**ENDIF**

**W_C = SQRT(ADD_C**2+PROP_C**2)**

**ERROR_P = W_P * ERR(1)**

**ERROR_C = W_C * ERR(1)**

**IPRED = IPRED_P**

**W = W_P**

**ERROR_TERM = ERROR_P**

**IF(DVID==2) THEN**

**IPRED = IPRED_C**

**W = W_C**

**ERROR_TERM = ERROR_C**

**ENDIF**

**IF (W.LE.0.000001) W=0.000001**

**IRES=DV-IPRED**

**IWRES=IRES/W**

**Y = IPRED + ERROR_TERM**

**IF (DVID==1.AND.ICALL==4.AND.Y<=LLOQ_P) Y=LLOQ_P/2**

**IF (DVID==2.AND.ICALL==4.AND.Y<=LLOQ_C) Y=LLOQ_C/2**

**IF(AMT>0) THEN**

**TIMEDOSE = TIME**

**AMOUNTDOSE = AMT**

**ENDIF**

**TAD = TIME-TIMEDOSE**

**IF(VISIT==1.AND.DVID==2) TAD = 25.5**

**IF(VISIT==2.AND.DVID==2) TAD = 15**

**IF(VISIT==3.AND.DVID==2) TAD = 15**

**IF(VISIT==4.AND.DVID==2) TAD = 15**

**VARCL = BSVCL + BOVCL**

**VARBIO = BSVBIO + BOVBIO**

**VARAUC = BSVBIO + BOVBIO - BSVCL - BOVCL**

**VARABS = BOVKA + BSVKA - BSVMTT - BOVMTT**

**AA1 = A(1)**

**AA2 = A(2)**

**AA3 = A(3)**

**AA4 = A(4)**

**$THETA (0,7.55,50) ; 1 CL_1 [L/h]**

**(0,79.0582,300) ; 2 V [L]**

**(0,6,10) FIX; 3 KA [1/h]**

**1 FIX ; 4 BIO**

**(0,0.188663,0.5) ; 5 PROP []**

**0 FIX ; 6 ADD [mg/L]**

**(0,0.514785,10) ; 7 MTT**

**(0,46.7027,300) ; 8 V3 [L]**

**(0,3.21354,50) ; 9 Q [L/h]**

**(0,1.70998,5) ; 10 SCALE_BOV**

**(-0.0616,0,1502) FIX ; 11 RTVAUC_CL**

**(-0.99,2.08781,10) ; 12 CL_2 [L/h]**

**(-0.99,1.01864,10) ; 13 CL_3/4 [L/h]**

**(0,0.480537) ; 14 BIO_VISIT2**

**(0,10,50) FIX ; 15 NN**

**(-0.99,-0.784495,10) ; 16 RIF_KA**

**(0,0.700687,10) ; 17 KE0**

**(0,0.647886) ; 18 PPC**

**(0,0.753099,1) ; 19 PROP_E []**

**(0,0,5) FIX ; 20 ADD_C [mg/L]**

**$OMEGA BLOCK(1)**

**0.0734386 ; 1 BSV CL**

**$OMEGA BLOCK(1) FIX**

**0 ; 2 BSV V**

**$OMEGA BLOCK(1) FIX**

**0 ; 3 BSV KA**

**$OMEGA BLOCK(1) FIX**

**0 ; 4 BSV BIO**

**$OMEGA BLOCK(1) FIX**

**0 ; 5 BSVV3**

**$OMEGA BLOCK(1) FIX**

**0 ; 6 BSVQ**

**$OMEGA BLOCK(1) FIX**

**0 ; 7 BSVV4**

**$OMEGA BLOCK(1) FIX**

**0 ; 8 BSVQ2**

**$OMEGA BLOCK(1) FIX**

**0 ; 9 BSVMTT**

**$OMEGA BLOCK(1) FIX**

**0 ; 10 BOVCL**

**$OMEGA BLOCK(1) SAME**

**$OMEGA BLOCK(1) SAME**

**$OMEGA BLOCK(1) SAME**

**$OMEGA BLOCK(1) SAME**

**$OMEGA BLOCK(1) SAME**

**$OMEGA BLOCK(1) SAME**

**$OMEGA BLOCK(1) SAME**

**;-------------------------**

**$OMEGA BLOCK(1)**

**0.237034 ; 14 BOVBIO**

**$OMEGA BLOCK(1) SAME**

**$OMEGA BLOCK(1) SAME**

**$OMEGA BLOCK(1) SAME**

**$OMEGA BLOCK(1) SAME**

**$OMEGA BLOCK(1) SAME**

**$OMEGA BLOCK(1) SAME**

**$OMEGA BLOCK(1) SAME**

**;-------------------------**

**$OMEGA BLOCK(1)**

**0.861212 ; 18 BOVKA**

**$OMEGA BLOCK(1) SAME**

**$OMEGA BLOCK(1) SAME**

**$OMEGA BLOCK(1) SAME**

**$OMEGA BLOCK(1) SAME**

**$OMEGA BLOCK(1) SAME**

**$OMEGA BLOCK(1) SAME**

**$OMEGA BLOCK(1) SAME**

**;-------------------------**

**$OMEGA BLOCK(1)**

**0.357417 ; 22 BOVMTT**

**$OMEGA BLOCK(1) SAME**

**$OMEGA BLOCK(1) SAME**

**$OMEGA BLOCK(1) SAME**

**$OMEGA BLOCK(1) SAME**

**$OMEGA BLOCK(1) SAME**

**$OMEGA BLOCK(1) SAME**

**$OMEGA BLOCK(1) SAME**

**;-------------------------**

**$OMEGA BLOCK(1)**

**0.0339448 ; BVVCL**

**$OMEGA BLOCK(1) SAME**

**$OMEGA BLOCK(1) SAME**

**$OMEGA BLOCK(1) SAME**

**$OMEGA BLOCK(1) FIX**

**0 ; 46 BSVKE0**

**$OMEGA BLOCK(1) FIX**

**0 ; 47 BVVPPC**

**$OMEGA BLOCK(1) SAME**

**$OMEGA BLOCK(1) SAME**

**$OMEGA BLOCK(1) SAME**

**$SIGMA 1 FIX**

**$ESTIMATION MSFO=run090_3.msf MAXEVAL=9999 PRINT=1 METHOD=1 INTER**

**NOABORT NSIG=3 NONINFETA=1 ETASTYPE=1 ;MCETA=1000 RANMETHOD=4P ; REPEAT**

**$COVARIANCE UNCONDITIONAL PRECOND=1 PRINT=E MATRIX=R**

**$TABLES;**
